# Supplementary figures and images for: NRF2 loss recapitulates heritable impacts of paternal cigarette smoke exposure
Source: PLoS Genet. 2020 Jun 10;16(6):e1008756. doi: 10.1371/journal.pgen.1008756 (PMC7307791; doi:10.1371/journal.pgen.1008756)

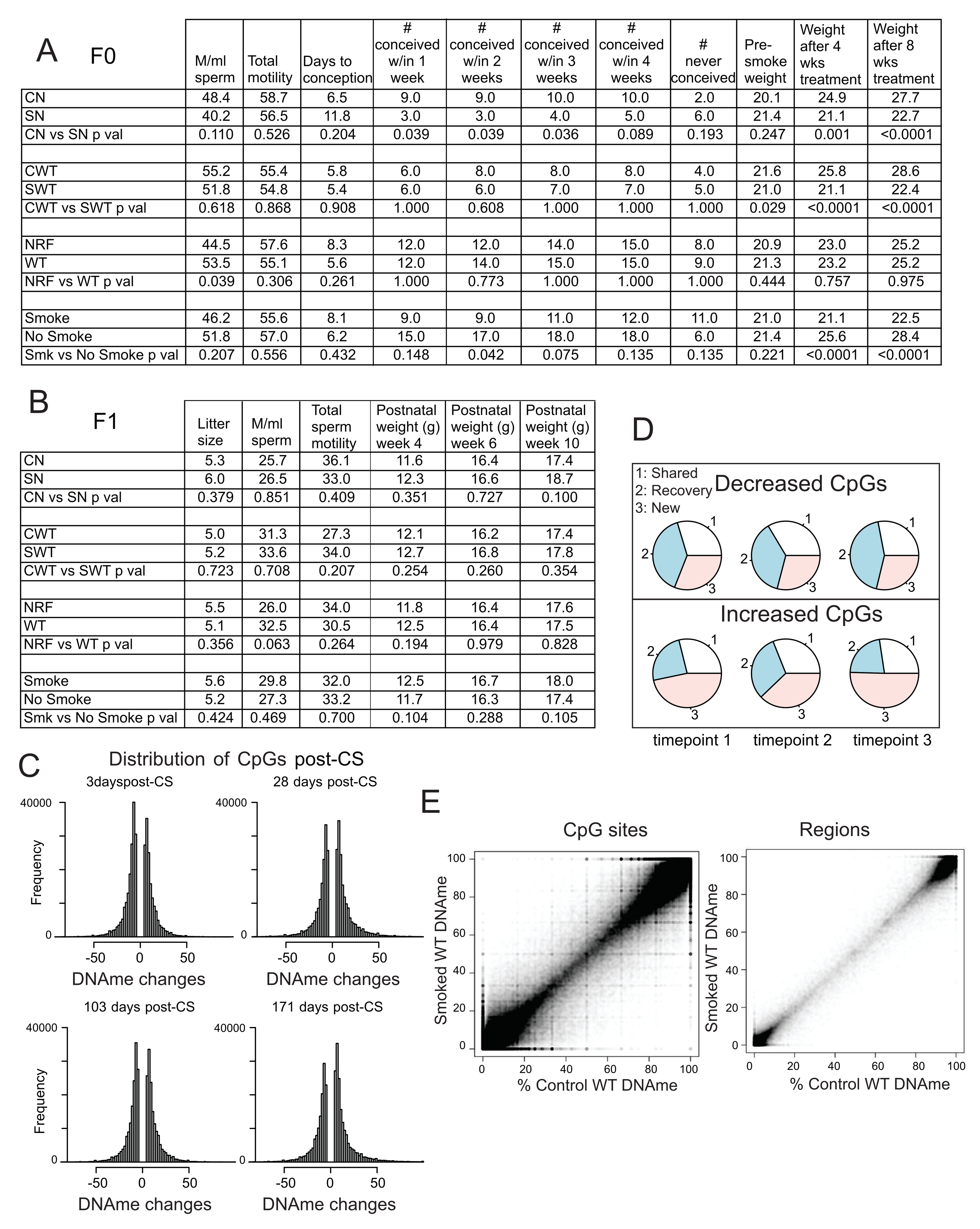

Supplement: S1 Fig — A) F0 animals- Both WT and Nrf2-/- mice that were exposed to CS weighed significantly less than non-exposed control animals. Sperm concentration and motility were not significantly impacted by CS exposure, sperm concentration was lower in Nrf2-/- than WT males, and conception was significantly delayed in CS-exposed Nrf2-/-mice compared with unexposed Nrf2-/-mice. B) F1 animals- Neither growth trajectories nor sperm parameters were different in F1 animals based on paternal CS exposure. Abbreviations: CN-control Nrf2-/-, SN-smoke-exposed Nrf2-/-, CWT-control wild type animals, SWT-smoke-exposed wild type animals. C) Quantitative data indicate that the number of differentially methylated loci in CS-exposed mice compared with age matched controls does not diminish following a recovery period of up to 171 days. D) When considering only loci that lost methylation in the CS-exposed group, about one third of differentially methylated loci persisted for the entire recovery period (white), one third returned to baseline levels (blue) and one third emerged as differentially methylated following a recovery period (pink). Contrastingly, for loci that gained methylation as a result of CS exposure, a smaller fraction of differentially methylated CpGs persisted or recovered while nearly half of differentially methylated loci emerged during the recovery period. E) As expected, the number of differentially methylated CpGs and the magnitude of change in DNAme was much higher than the changes observed regionally. (TIF) [file pgen.1008756.s001.tif]

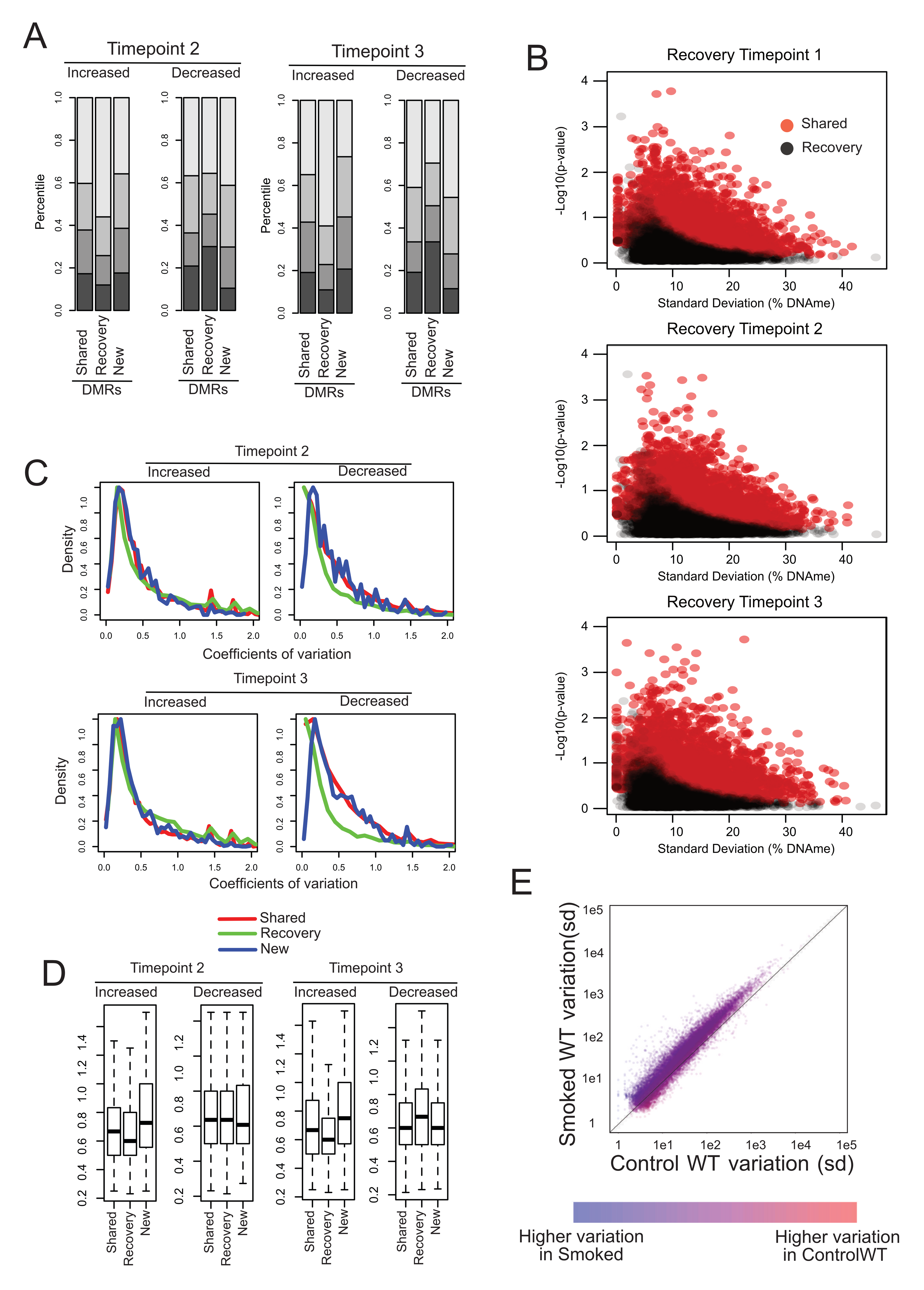

Supplement: S2 Fig — Summary of the recovery, persistence or emergence of differentially methylated loci (A and B) and properties of recovery regions (C, D and E) A) Impact of the initial methylation status and direction of change on methylation recovery. The hypomethylated DMRs (<25% DNAme) in which methylation increased and the hypermethylated DMRs (> 75% DNAme) in which methylation decreased with smoke exposure were more likely to recover. Regions displaying methylation between 25% and 75% Regions of intermediate DNAme were less likely to recover across both groups. Both comparisons were to the 3 days post-CS group. Timepoint 2 = 103-day recovery group, and timepoint 3 = 171-day recovery group. Both comparisons were to the 3-day recovery group. Dynamics were similar to those observed at 28 days post CS (see Fig 1D and 1F). B) DMRs where methylation changes were maintained across the recovery period tended to display higher overall variation and a higher degree of statistical significance compared with regions that recovered. C) In every category of DMR (shared, recovery or new) variation diminished as CpG density of a region increased. The impact of CpG density on variation was particularly apparent for regions in which DNAme decreased as a result of CS exposure and later recovered to baseline. D) DMRs that displayed increased DNAme in CS-exposed animals and later recovered were generally regions of lower CpG density, while DMRs that gained methylation and subsequently recovered were generally at regions of higher CpG density. E) Variation in F1 prefrontal cortex gene expression was significantly higher in CS-exposed animals compared with controls suggesting stochastic dysregulation of gene expression in paternal CS-exposed offspring and offspring of mice with reduced antioxidant capacity. (TIF) [file pgen.1008756.s002.tif]

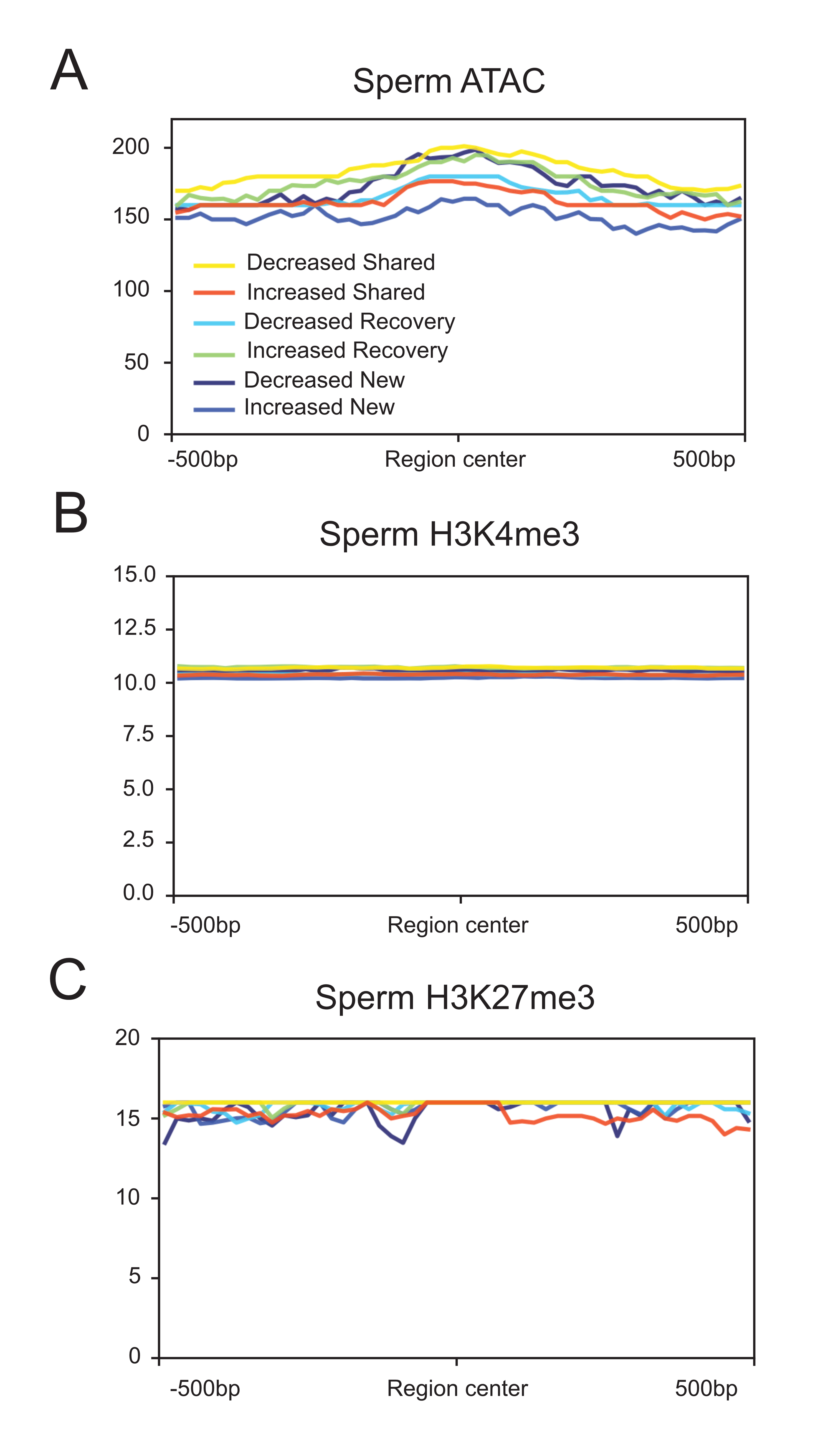

Supplement: S3 Fig — A) Chromatin accessibility was not predictive of the propensity for DMRs to recover or be maintained after removal of CS exposure. B-C) Localization of B) H3K4me3 and C) H3K27me3 was likewise not associated with DMR recovery or maintenance. (TIF) [file pgen.1008756.s003.tif]

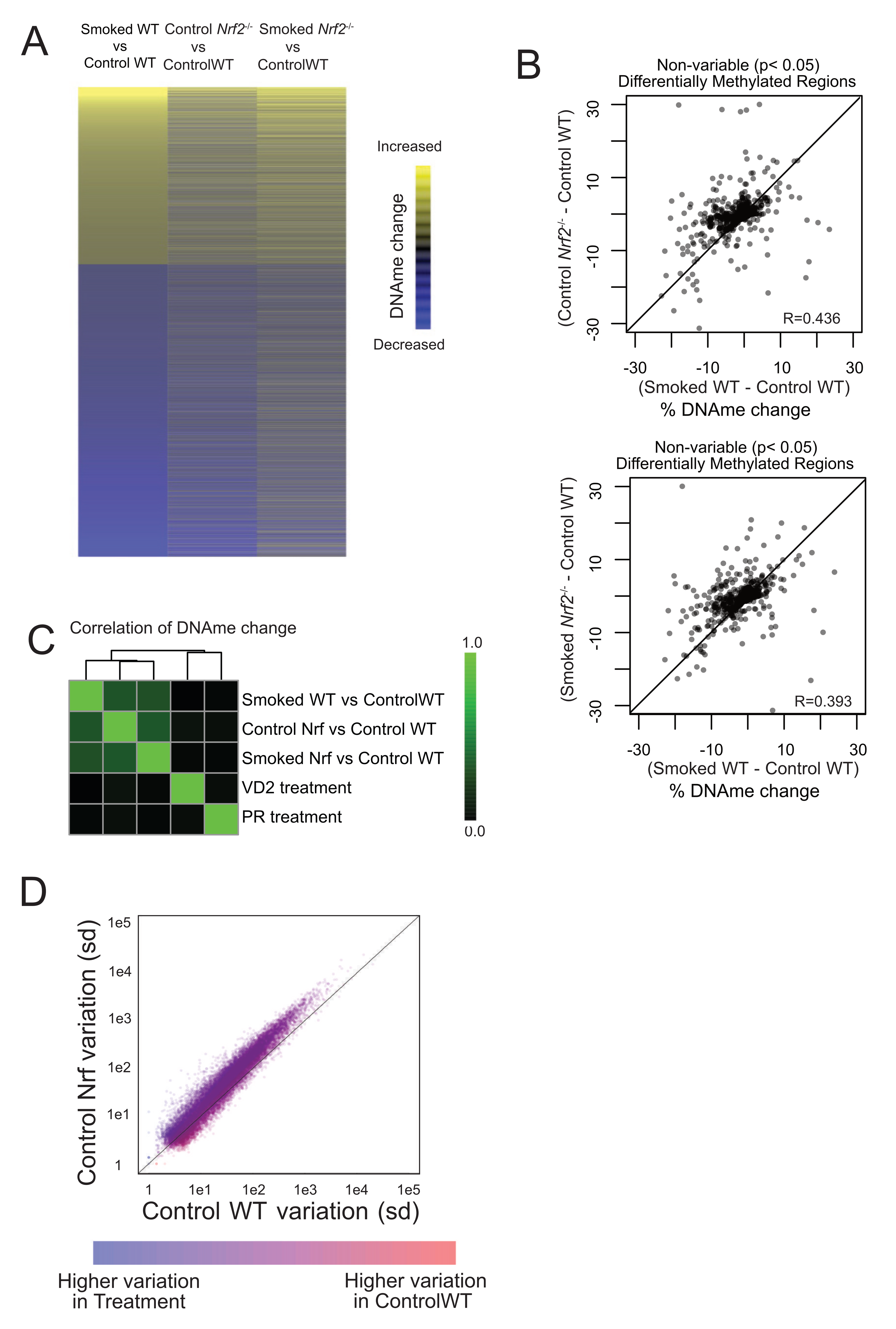

Supplement: S4 Fig — A) Heatmap illustrating the significant similarity between CS-associated sperm DMRs identified in WT mice and DMRs associated with the Nrf2-/- genotype, apparently independent of CS-exposure status. B) Association with CS exposure was maintained independent of the increased variation we observed in WT exposed mice C) A high correlation in DMRs was observed between CS-exposed WT mice and Nrf2-/- whether or not they were exposed to CS. No correlation was observed between DMRs identified in the current study compared with previously published DMRs associated with vinclozolin exposure (VD2) and protein restricted diet (PR). D) Variation in F1 prefrontal cortex gene expression was significantly elevated in Nrf2-/- controls compared with WT controls suggesting stochastic dysregulation of gene expression in offspring of mice with reduced antioxidant capacity. (TIF) [file pgen.1008756.s004.tif]
