# Supplementary material for: NRF2 loss recapitulates heritable impacts of paternal cigarette smoke exposure
Source: PLoS Genet. 2020 Jun 10;16(6):e1008756. doi: 10.1371/journal.pgen.1008756 (PMC7307791; doi:10.1371/journal.pgen.1008756)
Supplement: Attachment — Submitted filename: PlosGenetics_Smoking_Response_March2020.pdf [file pgen.1008756.s007.pdf]

DEPARTMENT OF BIOMEDICAL GENETICS

Patrick J. Murphy, Ph.D.
Assistant Professor of Biomedical Genetics

UNIVERSITY of ROCHESTER MEDICAL CENTER

Dear Drs. Bartolomei and Reik,

Thank you for your careful consideration of our manuscript and your critical assessment of our revisions. Along with my co-authors, we were quite happy that the reviewers were mostly satisfied with the changes we made in response to the initial reviews. We understand the additional requests from reviewers #1 and #3, and we now present a new revision of the manuscript where we accommodate these latest requests. Below we have listed these items, and beneath each item in blue text we indicate our response to the requests and the specific changes that were made to the manuscript.

Sincerely,

Patrick J. Murphy PhD
Assistant Professor
University of Rochester, Wilmot Cancer Institute

- Reviewer #2 is satisfied with our changes and makes no additional requests.

"The authors made satisfactory edits in response to the issues I raised."

- Reviewer #3 is mostly satisfied and makes one minor suggestion.

"I'm overall satisfied with the revision and recommend acceptance for publication in Plos Genet. I have only one more suggestions for consideration: Since cigarette smoke is a human activity and the authors have discussed the potential link with sperm tsRNAs, it would be good to include the recent papers about the acute dietary effect on human sperm tsRNAs and the related discuss on how this could be regulated by oxidative stress (Plos Biol 2019, PMID 31877125; Nat Rev Endocrinol 2020, PMID:32066893)"

We thank the reviewer for this suggestion, as it provided additional significance to our results. We have made the requested addition, and our changes can be found in the third paragraph of the discussion section, beginning at line 324.

- Reviewer #1 also seems mostly satisfied with our revision, as they make no direct suggestions for changes, but they do point out a key issue they would like us to address.

"the key shortcoming of replication (which looks like another referee raised) has not been addressed."

We thank reviewer #1 for further highlighting this important issue. In the newly revised manuscript we provide an additional paragraph, at line 349 in the discussion section, stressing the importance of replication, highlighting that the results of our study should not be over interpreted, and indicating that additional validation/replication of our study by an independent research group is required. We would also like to further address the issue noted by Reviewer #1 with the below comments.

Reviewer #1 is referring to comments from the initial review where they state that “the authors do not present an independent replication” and comments from Reviewer #2 stating “the molecular profiles have to be considered preliminary effects until such time they are validated.” In our revision we attempted to mitigate this concern in the discussion section. At Line 343 we state "...further studies are necessary to validate the current findings and to more fully explore the mechanisms underlying our observations." This textual addition to the manuscript was sufficient to satisfy Reviewer #2, as they now indicate that “The authors made satisfactory edits in response to the issues I raised.” Thus, only reviewer #1 still finds it necessary for us to address this issue further.

We would like to point out that we did indeed pay careful attention to replication during the design phase of this study, as well as during the data analysis phase. DNA methylation levels were quantified only at regions where we had sufficient read depth coverage for more than 4 biological replicates, and regions were excluded if any CpGs for a given region had read depths of 7 or fewer. Regions were also excluded if they contained fewer than 3 CpG, or if fewer than 4 biological replicates met these rigorous criteria. Importantly, the majority on regions analyzed had sufficient depth and CpG content for more than 8 biological replicates.

Beyond what we have already done, to ensure scientific rigor and attention to replication, it will be important that our entire study is replicated using a completely separate set of animals, treated under similar experimental conditions, using similarly rigorous high throughput genomics analysis methods. Such a study would be a tremendous undertaking. In order for such a study to be accomplished, it would also be very important that this type of study be conducted by a completely separate research group. This type of independent approach would eliminate any potential for confirmation bias and ensure that our results are indeed valid. We make note of this sentiment in the new paragraph of the revised manuscript. In the new revision, we also note that these types of independent replication studies “are particularly important in the epigenetics community, as several reports have questioned how frequently epigenetic inheritance occurs in mammals and humans”, and we also caution the reader against over interpretation of our results, indicating that “studies in mouse often to do not translate beyond the laboratory, and therefore, further epidemiological or clinical studies are required to measure the heritable impacts of oxidative stressors in humans.”

We believe these new additions to the manuscript and our detailed explanation for how we paid careful attention to scientific rigor and replication now satisfies the concerns of all reviews.
