# Supplementary material for: NRF2 loss recapitulates heritable impacts of paternal cigarette smoke exposure
Source: PLoS Genet. 2020 Jun 10;16(6):e1008756. doi: 10.1371/journal.pgen.1008756 (PMC7307791; doi:10.1371/journal.pgen.1008756)
Supplement: Attachment — Submitted filename: PlosGenetics_Smoking_Rev1_Response_Feb2020.docx [file pgen.1008756.s006.docx]

DEPARTMENT OF BIOMEDICAL GENETICS

Patrick J. Murphy, Ph.D.

Assistant Professor of Biomedical Genetics

Dear Editorial Staff,

We would like to begin by thanking the editors and reviewers for their efforts in helping us to publish this study. We found the reviewers to be thoughtful, fair, and critical. We found each of the reviewer’s comments and concerns to be very helpful during the revision process. We have now accommodated all reviewers’ concerns, and we are confident our manuscript has been significantly improved as a result. Below we have addressed each issue point-by-point, and our specific responses can be found beneath each noted concern highlighted in blue text.

Sincerely,

### Patrick J. Murphy PhD

### Assistant Professor

### University of Rochester, Wilmot Cancer Institute

**Reviewer 1 Concerns:**

1. It doesn't seem as if the methylation analyses have been analysed in a rigorous manner. For example, considering the initial sperm methylation differences in sperm (Figure 1), are any of the lists of sites significant at the genome-wide corrected level. I appreciate that P values such as 0.05 do not demarcate truth from falsehood, but it does give the reader some sense of what to make of the data. In this case it would be especially important as the authors do not present an independent replication. In fact, I can't find any description of the stats in the Methods either.

We thank Reviewer 1 for raising this issue, and we agree that rigorous analysis methods are critical for proper interpretation of DNA methylation data. For these studies we were highly rigorous, but a sufficient description of our analysis methods and the motivation behind our approach was not included in our prior manuscript. We have now corrected this, and performed additional analyses to ensure proper methods were implemented. In this regard, recent studies have found that increased variation in DNA methylation is a consequence of several environmental insults, including cigarette smoke exposure (Busche et al., 2015; Grundberg et al., 2013; Irvin et al., 2014; Petersen et al., 2014). Therefore, one goal of our study was to quantify variation in DNA methylation on a region-by-region basis and then compare variation levels between experimental conditions, previously depicted in Figures 1E & 2D. Because p-value-based thresholding methods exclude highly variable regions, thresholding-based approaches would have prevented us from making such comparisons. Instead, we quantified DNA methylation levels only at regions where we had sufficient read depth coverage for more than 4 biological replicates, and regions were included only if all CpGs for a given region had sequence read depth of 8 reads or more. If any CpG within the region lacked sufficient read depth, if the region contained fewer than 3 CpG, or if fewer than 4 biological replicates met these criteria, those regions were excluded. In most cases more than 8 biological replicates met these criteria. Once scoreable regions were identified, we assessed changes in DNA methylation, the inter-replicate variation in DNA methylation level, and the statistical significance of DNA methylation changes (post-hoc 2-sample t-tests).

A better description of this approach and rationale is now included in the results section and additional figure panels are included in Supplemental Figure 2. Additionally, when we restricted our analysis to only statistically significant changes, excluding the highly variable regions, we confirmed our results that methylation changes in smoked mice are similar to changes in NRF2 null mice. These data are now included in Supplemental Figure 4.

2. In Figure 3, the authors say that CS induced meth changes in F0 sperm are very similar to those found in the NRF F0 sperm. But they only present heat maps. This needs to be redone as scatter plots just like they do for F1 mice in Figures 3B-D. On that note, these latter three analyses (B, C and D) are the most convincing and interesting in this manuscript.

We thank the reviewer for this suggestion and have made the appropriate figure adjustments. The F0 data is now in scatterplot format in Figures 3A-C, along with the F1 data in Figures 3D-F. We have moved the heatmap from Figure 3 to Supplemental Figure 4A.

3. If I am not mistaken, there are studies showing the impact of smoking on sperm in humans. Can the authors perform some comparisons using the published data? If there are similarities it would certainly strengthen the case.

Indeed, Reviewer 1 is correct, there are studies measuring the impacts of cigarette smoke exposure in humans, and we are authors for one of these studies (Jenkins et al. Andrology 2017). We also agree that it would be quite informative if we were able to compared the results of these two studies, and we attempted to do so. Here, we compared our mouse RRBS data with the methylation array-based data from the human studies. Unfortunately, this attempt failed because neither method (RRBS or array-based) provides genome-wide coverage, and each method selects for different genomic regions. We found almost no overlap in the scorable regions comparing RRBS with the methylation array, and differences in the mouse and human genomes prevented us from directly comparing across genomes for the few regions that might have otherwise been scorable. Thus, we find that the analysis requested by Reviewer 1 is not possible, unfortunately.

4. In F1 brains, was there any correlation between methylation and expression?

We thank Reviewer 1 for this suggestion, and thus performed additional analysis to investigate the relationship between DNAme and gene expression in the F1 brains. Here, when comparing F1 brain of the smoked mice to that of the control mice, we identified 28,707 regions that display greater than 5% DNAme changes, and 134 genes that are significantly differentially expressed (|log2(fold change)|>1 and p-value < 0.05). To determine whether the observed gene expression changes were related to differential methylation we intersected DMRs located within 1kb of transcription start sites (TSS) with differentially expressed genes. To calculate the p-value for enrichment, we utilized the BedTool ShuffleBed function, to randomly select 134 TSS regions, and intersected these DMRs, with 1000 total permutations. We found only 7 DMRs within 1kb of a TSS that intersected differentially expressed genes (p-value = 0.1). We were unable to conclude that DNAme change influenced gene expression in the prefrontal cortex, likely due to the dramatically different coverage of RRBS data compared with RNAseq data. We have added these analyses and results to the manuscript.

5. I think in the discussion they should list the variety of shortcomings of their study. This in no way weakens their message but rather provides a more balanced view. For example, the bar now in this field is it perform IVF using sperm isolated from exposed F0 males so that the F0 males and females never come into physical contact (and eliminates other effects such as those potentially mediated by olfactory systems).

We appreciate this point raised by Reviewers 1 and 2 and have discussed this and other limitations of the current study in the second-to-last paragraph of the discussion section.

**Reviewer 2 Concerns:**

Major Issues:

The conclusion that the paternal CS exposure "underlies" offspring molecular profiles is not sufficiently supported by the design and analyses. Throughout the manuscript, the authors use the language that the Nrf2 knockout mouse data "largely" recapitulate the effects seen in CS exposed sperm and offspring tissue. Largely implies a preponderance of signal that is shared. I agree that there is compelling overlap, but not that the it is recapitulated largely. Blocking the effect of paternal CS in F0 sperm or F1 somatic tissue with co-administration of antioxidants (e.g. tocopherol, or NAC) would support this mechanistic claim. Additionally, validating that there is indeed oxidative stress in the paternal germ cells or offspring brain (and that it is reflected similarly in the Nrf2-/- model) could provide additional direct support. In the absence of this, the claims have to be toned down throughout the manuscript, and the title (which currently implies causally supported results) and section headings of results sections would have to be modified considerably. For example, the section header on line 219 is particularly liberal with respect to the data.

We thank Reviewer 2 for making note of this issue. Indeed, we do not make direct measurements of oxidative stress, or administer antioxidants to test mechanism of oxidative stress. We therefore understand that our results might be mis-interpreted, and so we have adjusted the text accordingly. Notably, the *Nrf2-null* model is an established system for studying the downstream consequences of oxidative stress, and this genetic system is commonly used across several model organisms. If we were studying mechanisms of oxidative stress, we would certainly perform the additional experiments suggested by Reviewer 2, but since our study only measures consequences of oxidative stress, we are confident that our approach and conclusions are justified. To further accommodate these concerns, we have made several textual changes to the manuscript to moderate our conclusions, and we clarify that in our mouse study loss of *Nrf2* is used as a model to study oxidative stress outcomes.

The design of the study and the manuscript do not acknowledge paternal-independent effects on offspring molecular phenotypes. Dams may detect the exposure and invest differentially (e.g. PMID: 29514964) during gestation or lactation, which could contribute to offspring brain and sperm molecular signatures. In other words, the CS exposed males could represent poorer quality mates, or simply have altered olfactory signals, which leads the dams to invest less in the progeny. The only way(s) to account for this are to perform IVF and embryo transfer and/or perform cross-fostering to account for all maternal influences, and post-natal influences, respectively. I acknowledge that those experiments are often not feasible, so in such cases this possibility must be carefully acknowledged. Similarly, offspring sex and litter sex ratios have not been disclosed and should be. Alterations to sex ratios could alter molecular signatures in brain and sperm in F1.

We thank Reviewer 2 for this comment. An important detail that we inadvertently omitted from the manuscript is that RNAseq and RRBS of prefrontal cortex tissue were performed exclusively on male offspring (in order to avoid confounding due to sex-related differences). We have updated the manuscript to clarify this point. We have also indicated that sex ratios in F1 litters did not differ significantly between groups, and have updated the discussion section to discuss limitations of the study (as suggested by Reviewer 1 as well), including include the possibility that dams may invest differentially in the offspring of smoke-exposed sires.

Other Issues/Questions:

Was there any attempt to compare the mouse CS exposure DNAme and RNA-seq signatures to the previous human sperm publication data (e.g. the cited Jenkins et al., 2017 Andrology work)? If not, why?

We agree that performing this type of analysis would be quite valuable, and we attempted to make these comparisons. However, we found this analysis approach was not possible due to incompatibility in DNA methylation measurement methods. Please refer to our response to Reviewer 1 Point #3 for additional details.

RNA-seq data from prefrontal cortex samples are repeatedly listed as "neural" signals (abstract, line 35; pg 9 line 351), but the samples represent mixed cell types from bulk tissue, not just neurons.

We have corrected the text to be more accurate in this regard.

Data from GSEA enrichment from ranked RNA-seq only include the two (Oxphos and ROS) (Fig 2E). These are certainly significant enrichment scores, but where do they rank amongst all MSigDB gene sets? Without the data on the other sets, your emphasis of those two could be seen as "cherry picking." A supplemental table listing all significant Gene sets, NES scores, and p-values (positive or negative enrichment) would provide appropriate transparency.

We have added this supplemental table, and thank Reviewer 2 for this suggestion.

I do not feel that global gene expression variability should be interpreted as an effect or outcome. I recommend focusing on the enrichment and ontology analyses instead.

We have de-emphasized the increased variation in gene expression as an outcome and instead focus in the enrichment and ontology data, as suggested by Reviewer 2. However, we have highlighted the literature mentioned by Reviewer 3 with regards to epigenetic variability related to various environmental exposures.

There are no validation experiments to validate DMRs (e.g. targeted bisulfite seq) or differentially expressed genes (e.g. qPCR) between treatment groups and/or genotypes. Accordingly, the molecular profiles have to be considered preliminary effects until such time they are validated.

We thank Reviewer 2 for raising this issue. Currently, RRBS and RNA-seq studies have become so frequent in the epigenetic community that these methods are now considered established approaches for measuring DNA methylation and gene expression levels. Very rarely do current studies perform targeted BS-Seq or qPCR methods for validation. However, as Reviewer 2 suggests, we have added text to the discussion section indicating that additional studies are necessary in order to independently validate our conclusions.

The statement on page 5 in the second paragraph (line 183) stating that recovery influences suggest CpG density has something to do with buffering environmental influences is a bold statement. It is an interesting idea, but perhaps it is safer to say that DNAme across CpG regions may buffer environmental influences? You may need to clarify what you mean better, provide some sort of support, or move it to the discussion with appropriate level of language stating that is speculative.

We thank Reviewer 2 for their concern about this statement and agree it is quite bold. We have therefore moved this comment to the discussion and adjusted our language, as suggested by the reviewer.

Figure S3 panel D should have labels on the x-axis as well.

Thank Reviewer 2 for pointing out this error. We have ensured that all figures include the appropriate labels.

Complete enrichment score data from GSEA are not present.

This was a mistake in our initial submission. We thank the Reviewer 2 for noting this, and the table is now included.

**Reviewer 3 Concerns:**

1. First and foremost, the abstract should be reconstructed to be more informative on the major findings and to be more accurate on the results. For example, the long sentence “This study used mouse models to evaluate: 1)…2)…3)…4)…” is not fully necessary and can be reduced or removed, the saved space could be used to describe more results.

We thank Reviewer 3 for this comment and suggestion. We have modified the abstract in the revision to better describe the major findings of our study.

The sentence “paternal smoking causes changes in neural DNAme and gene expression in offspring” is not accurate and potentially misleading, as the changes in F1 neural DNAme and gene expression in offspring, in fact, shows minimal overlapping with the paternal CS-associated DMRs in in smoke-exposed F0 sperm. This is an important piece of information and should be clearly stated in the abstract or it would be misleading.

We thank Reviewer 3 for pointing out this potential misleading statement. While we agree that there is minimal overlap in the paternal CS-associated DMRs in smoke-exposed F0 sperm compared with the F1 prefrontal cortex DNAme, we do in fact find that paternal CS-exposure is associated with DNAme changes in offspring. The mechanism by which these changes occur is unknown, and based on our result it is unlikely a direct result of paternal sperm DNAme levels. We have clarified this description in the results to prevent misinterpretation.

The author also didn’t find overlapping between the DNAme between F0 and F1 sperm, which is consistent with the previous finding in other mammalian epigenetic inheritance models (Science 2014, PMID: 25011554; Genome Biol 2015, PMID: 25853433), and this result should be highlighted in the abstract. In fact, these results suggest DNAme may not be a direct vector in transmitting epigenetic phenotypes (it could be a marker representing changes in sperm epigenome but could be secondary effect), and his has triggered the emerging studies that focusing on sperm RNAs as the epigenetic information carriers in the transmission of phenotype, as extensively discussed recently (Nat Rev Genet 2016, PMID:27694809; Nat Rev Endocrinol 2019, PMID: 31235802). These should be discussed in the manuscript to make an informative paper.

We agree with Reviewer 3 that F0 sperm DNAme might be best defined as a marker of epigenetic inheritance rather than a direct facilitator of inheritance. We attempted to make this point in the discussion of our initial submission, and now in the revised manuscript we have better emphasized this point both in the abstract and in the discussion. We have also highlighted studies describing how sperm tsRNAs might be a functional contributor to epigenetic inheritance.

2. In the results, the authors showed that “Interestingly, paternal CS exposure caused a globally elevated variation in gene expression in the F1 brains (Figure 2A)”, this is an interesting finding and may intricately related to epigenetic mechanisms. I suggest the authors to read recent publications on the case of Trim28 (Genome Biol 2010, PMID: 21092094; Cell 2016 2016, PMID: 26824653) which may shed new lights on the current study and should be at least discussed. The author may also check their DNAme and RNA-seq data regarding the status of Trim28. Did the authors find any correlation between F1 brain DNAme and transcriptome changes? This point is not clearly stated. If the conclusion is negative, relate discussions should be provided. There could be many explanations.

We thank Reviewer 3 for bringing these studies to our attention, and we have added this interesting work to the discussion. We did not see differential expression of *Trim28* or *Dnmt3a* in our studies, but this seems to be an important and potentially relevant mechanism for the signals we observed. We have also attempted to compare our F1 DNAme data with our RNA-Seq data, as suggested by Reviewers 1 and 3, but these comparisons were not possible. Please see our comment to Reviewer 1 point #4.

3. The effect of NRF2 deletion on F0 sperm and F1 brain that mirrors the CS-exposure is striking, this could be due to oxidative stress and it raise many interesting questions. The effect could involve additional epigenetic changes in the sperm if the oxidative stress is the main effector. For example, oxidative stress is a strong inducer of tRNA cleavage and will generate more tsRNA and rRNA fragments (RNA 2018, PMID:18719243), and both tRNA and rRNA fragments (tsRNAs and rsRNAs) have been involved in sperm mediated intergenerational epigenetic inheritance of phenotypes (Science 2016, PMID:26721680; Science 2016, PMID:26721685; Nat Cell Biol 2018, PMID:29695786). These are interesting clues can be discussed in the paper.

We thank Reviewer 3 for this comment and have incorporated this in the discussion. Indeed, it seems RNAs, including tRNAs, might explain much of our observations, as Reviewer 3 notes. We now better articulate this in the revised manuscript.
